# Improving long term outcome for diabetic patients undergoing surgical revascularization by use of the radial artery conduit: a propensity matched study

**DOI:** 10.1186/1749-8090-8-27

**Published:** 2013-02-19

**Authors:** Darryl M Hoffman, Kamellia R Dimitrova, Helbert DeCastro, Patricia Friedmann, Charles M Geller, Wilson Ko, Robert F Tranbaugh

**Affiliations:** 1 Division of Cardiac Surgery and Office of Grants and Research Administration, Beth Israel Medical Center, New York, NY 10003, USA; 2 Present address: Department of Surgery, Montefiore Medical Center, Bronx, NY, USA; 3 Beth Israel Medical Center, First Avenue at 16th Street, New York, NY 10003, USA

**Keywords:** CABG, Radial artery graft, Diabetes, Propensity match

## Abstract

**Background:**

Diabetes predicts worse outcomes after coronary artery bypass grafting (CABG) We hypothesized that a strategy using radial artery (RA) conduit(s) would improve outcomes and long term survival for diabetic patients undergoing CABG with Left Internal Thoracic Artery (LITA) and RA grafts, with or without additional saphenous vein (SV) when compared with outcomes for patients bypassed with LITA and SV but no RA.

**Methods:**

A propensity matched study of long term survival in diabetic patients who had isolated first time CABG from January 1995 to June 2010 at an urban academic medical center in New York City. Our primary endpoint was all cause mortality determined from the Social Security Death Index in December 2010.

**Results:**

We compared our 15 year outcomes in diabetic patients after isolated, primary CABG: 642 patients received LITA + RA +/− SV (RA group) vs. 1201 patients who had LITA + SV only (SV group). Propensity scoring for multiple preoperative and operative variables matched 409 patients from each group: 68% were male with an average age of 61 years and ejection fraction averaged 47%. Average grafts per patient was 3.7 for both groups with 2.3 arterial grafts per patient for the RA group. Operative (30 day) mortality was 0.1% RA vs. 1.9% SV, (p<0.0001) For propensity matched patients, mortality was 0.25 RA vs 0.5% SV. (p<0.001) The incidence of major complications was similar in both groups. Kaplan Meier actuarial survival at 1, 5, 10 and 12 years was 98%, 89%, 77 and 70% for RA vs. 96%, 87%, 64% and 59% for SV (p<0.006.) By Cox multivariate analysis significant predictors of mortality were: age, stroke, peripheral vascular disease, COPD, creatinine > 2.5mg/dl and low ejection fraction but only RA use predicted better survival [HR 0.683, CI 0.507- 0.920, p=0.0122].

**Conclusion:**

For diabetic patients having CABG with LITA, use of radial artery conduit adds a substantial and sustained survival advantage compared to LITA and vein. Optimal revascularization for diabetics with multi vessel disease is redefined.

## Background

Surgical revascularization (CABG) remains the best evidence based treatment for atherosclerotic multivessel coronary artery disease in patients with diabetes, despite improvements in percutaneous interventional techniques (PCI) and medical therapy. The Achilles heel of PCI, especially in diabetics, is still the more frequent need for reintervention in the first years after the procedure [1]. In the long term, CABG too is vulnerable, because, while excellent and sustained long term patency of LITA grafts have been reported beyond 20 years, vein graft failure rates approach 30% at 10 years and 68% at 15 years [2,3].

Our group shares the usual concerns in diabetic patients about using both internal thoracic arteries which can compromise blood supply of the sternum and interfere with wound healing. In diabetic patients, all outcomes after CABG are known to be worse [4]. Failure of saphenous vein grafts is known to occur more often in diabetics [5], and we postulated that such vein graft failure might reduce long term survival in these patients. In our observational series [6], of over 1,500 consecutive isolated CABG patients, those receiving a radial artery (RA) graft, whether diabetic or not, experienced the same excellent and sustained medium to long-term survival. We aim in this present study to examine this important observation in detail and test the hypothesis that use of the RA as a second arterial conduit improves long term survival after CABG for diabetic patients.

## Methods

The Division of Cardiac Surgery at the Beth Israel Medical Center maintains a prospectively collected database on all patients undergoing cardiac surgery as required for the mandatory and audited New York State Department of Health Cardiac Surgery Reporting System. All data are reported to the State and also are maintained in a parallel, separate database. This study was approved by the Institutional Review Board, which waived written informed consent. Analysis of these data was retrospective

Our study population was drawn from the 4,500 patients who underwent first-time isolated CABG at our center from January 1995 to June 2010. From this cohort, the 1843 consecutive diabetic patients were selected to form the study group for this current analysis. Throughout the study period the New York State Cardiac Surgery Reporting defined diabetes as any patient on either oral hypoglycemic drugs or insulin prior to hospital admission for CABG.

In all these patients the LITA was grafted to the LAD, and additional grafts were constructed with SV or RA as necessary. Throughout the study, all SV harvest was endoscopic; RA harvest before 2000 was open and exclusively endoscopic since mid 2000. Our indications for using a RA graft were: age less than 60 years in the early years and then more liberally; unavailable or unsuitable SV accounted for our RA use in older patients. RA were used to bypass target arteries affected by stenosis of at least 70% usually in the left sided circulation. Patients on or likely to need hemodialysis were excluded to preserve potential dialysis access.

Throughout the period covered by this study, strict control of blood glucose level post CABG (target level 80–120 mg/dl) was protocol driven and supervised by active oversight from the Diabetes team comprising Endocrinologists in training and Faculty from the Friedman Division of Endocrinology. The same team transitioned patients from continuous infusion insulin to a personalized regimen for glycemic control.

### Surgical and radial artery harvesting technique

Details of RA harvest and surgical technique have been published elsewhere. (5) Over the study period RA use increased from ½ to 2/3 of all our CABG patients. In brief, operations were conducted under general anesthesia, on pump using cold blood cardioplegic arrest and a single cross clamp technique. RA grafts were generally used as aortocoronary bypasses; proximal anastomosis to another RA, SV or very rarely, LITA was made only when lack of RA length or aortic disease precluded direct aortic anastomosis. Off pump procedures (usually for extensive aortic disease) were performed in 48 (2.6%) of all patients (3.3% of the SV patients and 1.2% of the RA patients) but none of the propensity-matched patients had off pump surgery.

Harvest of RA was undertaken by our Physician Assistant or RNFA after Allen‘s test and pulse oximetry confirmed adequate collateral circulation. Less than 2% of evaluated RA were not harvested. No patient suffered postoperative hand ischemia. An open no-touch technique was used for harvest until 1999; thereafter, and building on extensive experience with endoscopic vein harvest, endoscopic RA harvest was performed with a Harmonic scalpel. After 2000, harvest was exclusively endoscopic and there has not been a single conversion to open technique. We administer intravenous Diltiazem after induction of anesthesia and postoperatively switch to oral nitrates or Diltiazem (ideally maintained for at least 6 months). All patients received aspirin, statins, beta-blockers and ACE inhibitors postoperatively (unless contraindicated).

The harvested RA was stored until use in a solution of the patient’s heparinized venous blood (containing circulating Diltiazem) mixed with 1% Papaverine. While RA from diabetic patients for the most part are excellent conduits, we have found occasional areas of minor calcification and thickening [7]. A rare harvested RA had to be discarded because of unsuspected intraluminal disease.

### Statistical methods

Statistical analysis was performed with the SAS system v9.2 (SAS Institute, Cary NC). Continuous variables are expressed as mean and standard deviation, and were analyzed using the t test. Categorical variables are expressed as percentages and were analyzed using the Chi Square and Fisher’s exact tests. The primary endpoint in this study was all cause mortality obtained from the Social Security Death Index (searched in December 2010). Patient survival was estimated by the Kaplan Meier method, and the log-rank test was used to assess survival differences between groups. Hazard ratios and 95% confidence intervals are presented. Cox regression was used to assess independent predictors of survival.

### Propensity matching

After excluding 47 patients on hemodialysis (1 RA and 46 SV) and 29 patients who died within 30 days after CABG, 1767 patients were subjected to propensity matching using the sequential nearest neighbor selection [7]. Propensity matching employed the following variables: age, gender, year of surgery, surgeon, ejection fraction, hypertension, transmural myocardial infarction, stroke, carotid disease, peripheral vascular disease, hemodynamic instability at time of procedure, current CHF, COPD, extensive ascending aortic atherosclerosis, extent of coronary artery disease, number of grafts per patient, and serum creatinine level > 2.5 mg/dl.

We used these variables as predictors in a logistic regression model – with radial artery use as the dependant variable. The output of this logistic regression model generated propensity scores for each patient that were then used to select pairs from among RA and SV patients, where each pair had probability scores differing by no more than 1%. 818 patients were included as we matched 409 patients from each of the two groups (RA and SV).

## Results

We studied in retrospect the 1843 consecutive diabetic patients, who underwent isolated, primary CABG in our institution January 1995 - June 2010. Of these, 1201 patients were bypassed with a LITA graft and SV, as needed, (GROUP SV) while 642 patients were grafted with LITA and RA with additional SV as needed (GROUP RA).

Table 1 summarizes the preoperative risk factors, and Table 2 the operative and postoperative data for all 1843 patients. Briefly, the patients grafted with RA were younger (mean age 58.6years ±8.1 vs 67.3 ±8 years), mostly male (72.6 vs 67.3%) and had fewer comorbidities. Preoperative vascular disease, especially carotid disease was substantially more prevalent before matching in the SV group, but interestingly not previous stroke. Single-vessel disease was uncommon, and left main stenosis was present in 30%. Reflecting our hospitals location and the communities it serves, surgical priority was strongly tilted to urgent surgery, (i.e. within the same hospital admission). Less than 25% of our patients had elective surgery; fewer than 5% had emergency surgery. Almost no patients in the RA group were hemodynamically unstable: despite the dexterity of our harvester, RA harvest adds 20–30 minutes to the procedure prior to sternotomy.

**Table 1:** Diabetic patients before propensity matching

	**Radial n=642**	**Vein n=1201**	**P value**
All-cause mortality	15.42%	38.13%	<0.0001
Mean age, years	58.6 (±8.1)	67.3 (±8.8)	<0.0001
Males	72.59%	59.03%	<0.0001
Hispanic	29.75%	28.06%	0.4443
Race			
White	62.15%	60.28%	0.517
AA	11.99%	16.65%	0.003
Other	25.86%	23.06%	0.201
Mean EF	48.3% (± 11.8)	45.9% (±13.5)	<0.0001
Years since surgery	6.7 (±3.9)	6.9 (±4.5)	0.257
BMI	29.8 (±5.5)	28.8 (±5.4)	<0.0001
Transmural MI	31.62%	36.97%	0.0219
Stroke	8.10%	9.91%	0.2023
Carotid disease	7.31%	17.24%	<0.0001
Aortoilac disease	2.02%	4.16%	0.0161
Femoral PVD	8.72%	12.99%	0.0062
Hemodynamics unstable	0.93%	1.67%	0.2051
Hypertension	71.18%	79.60%	<0.0001
Current CHF	6.39%	12.16%	<0.0001
COPD	20.72%	32.47%	<0.0001
Ascending aortic disease	3.12%	8.99%	<0.0001
Creatinine > 2.5 mg/dL	1.87%	7.16%	<0.0001
Coronary vessel disease			
Triple	86.2%	81.5%	0.0001
Double	11.25%	13.47%	0.0409
Single	2.05%	3.74%	0.0002
Left main	27.03%	31.4%	0.0017

**Table 2:** Diabetic patients before propensity matching

	**Radial n=642**	**Vein n=1201**	**P value**
Mean cross-clamp	71.8 (±20)	61.6 (±21)	<0.0001
Mean perfusion	94.8 (±25)	85.4 (±29)	<0.0001
Grafts/patient	3.84 (±0.9)	3.56 (±0.8)	<0.0001
Priority			
Elective	22.8%	19.12%	0.0100
Urgent	72.97%	75.15%	0.2370
Emergent	4.2%	5.74%	0.4400
Operative mortality	0.2%	1.9%	<0.0001
Permanent stroke	0.93%	1.25%	0.0059
Perioperative MI	0.67%	0.78%	0.6548
Sternal infection	1.4%	2.75%	0.2506
Septicemia	1.71%	3.66%	<0.001
Reop. for bleeding	1.71%	2.16%	0.1694
Respiratory failure	2.665%	5.25%	<0.0001
Renal failure	0.93%	2.91%	<0.0001
Hemoglobin A1C	8.1 (±2.2)	6.9 (±1.0)	0.005

The distribution of coronary artery disease was quite similar but there was a slight but significantly greater incidence of triple vessel disease (86.2% vs 81.5%) in the RA group. None of the patients in the cohort had bilateral ITA grafts. We very rarely use the LITA as a sequential graft and in diabetic patients avoid bilateral ITA harvest. In keeping with our intent to achieve complete arterial revascularization of the left coronary artery system, 35% of RA patients received more than 2 arterial grafts, mostly by using the RA as a “manufactured” Y graft. Bilateral RA grafts were harvested from 58 patients (9%) Grafts per patient averaged 3.7 for all patients; RA patients received on average 2.3 arterial grafts but SV patients received exactly 1.0 arterial graft per patient. Saphenous vein grafts were generally preferred over RA to bypass the right coronary system (almost always to the Posterior Descending artery).

Outcomes and complications for all diabetic patients are reported in Table 2. Briefly, outcomes were better in the RA group (RA operative mortality 0.1% vs 1.9% in the SV group, p<0.0001), and major complications were significantly less common in the RA group vs SV (Stroke 0.9% vs 1.25%, p=0.0059; Renal failure 0.9% vs 2.9%, p<0.0001; Respiratory failure 2.65% vs 5.25%, p<0.0001). Of note, only 2 significant infections occurred in the forearm and no major peripheral neurologic injury occurred.

Even before matching the mean duration from surgery to study was similar for the two groups at just over six and half years. After propensity matching, long-term follow-up was similar for RA matched patients at 6.7 ±3.9 years (median 6.49 years) and for SV matched patients 6.9± 4.5 years (median 6.88 years).

Hemoglobin A_1_c levels as an index of diabetes control was significantly different and actually worse for the RA group (8.1± 2.2 vs 6.9 ± 1.0; p =0.005).

### Outcomes in the propensity matched patients

Table 3 summarizes the preoperative risk factors for the RA and SV patients after propensity matching. Our hospital serves many New York immigrant communities and racial diversity is wide. “Other” in our hospital are mostly Asian. Reduced RA use in African Americans reflects the high incidence of hypertensive renal dysfunction in this group. Our bias is to avoid RA harvest in patients with renal dysfunction to preserve possible dialysis access.

**Table 3:** Groups after propensity matching; preoperative data

	**Radial n=409**	**Vein n=409**	**P value**
All-cause mortality	18.8%	25.4%	0.0230
Mean age, years	62.4 (±7.4)	63.4 (±7.9)	0.1111
Males	67.24%	68.95%	0.5995
Hispanic	31.05%	28.85%	0.4921
Race			
White	62.10%	56.97%	0.2470
African American	12.71%	16.14%	0.4030
Other	25.18%	26.89%	0.8620
Mean EF	47.1 (±12.5)	48.5 (±13.7)	0.1710
Years since surgery	6.5 (±4.2)	6.4 (±3.7)	0.3955
BMI	29.8 (±5.6)	28.6 (±5.7)	0.8130
Transmural MI	33.50%	31.78%	0.6017
Stroke	8.31%	8.56%	0.8999
Carotid disease	8.56%	7.82%	0.7021
Aortoilac disease	2.93%	4.16%	0.3445
Femoral PVD	10.02%	11.00%	0.6484
Hemodynamics unstable	0.73%	1.22%	0.4773
Hypertension	73.84%	73.35%	0.8740
Current CHF	8.07%	7.09%	0.5972
COPD	24.45%	24.225%	1.0000
Ascending aortic disease	4.16%	3.42%	0.5828
Creatinine > 2.5 mg/dL	1.96%	1.96%	1.0000
Coronary vessel disease			
Triple	79.4%	82.5%	0.7790
Double	16.5%	13.12%	0.5960
Single	3.1%	3.4%	0.9710
Left main	30.5%	29.7%	0.9065

Propensity-matched patients show strikingly similar demographics and risk factors summarized in Table 3. Important operative details of propensity matched groups are reported in Table 4: while aortic cross clamp time and perfusion time each averaged 10 minutes longer for the unmatched RA group (p<0.0001), after propensity matching, the differences between RA and SV groups remain significant only for cross clamp time. Perhaps reflecting our philosophy on complete revascularization, RA patients received slightly more grafts per patient, but after propensity matching, grafts per patient averaged 3.7 for both groups. For the propensity matched patients, there were no deaths in either group (hospital mortality 0% RA vs. 0% SV ; p=1).

**Table 4:** After propensity matching: operative data/complications

	**Radial n=409**	**Vein n=409**	**P value**
Mean cross-clamp	70.5 (±19.8)	66.7 (±17.6)	0.0110
Mean perfusion	93.9 (±24.7)	91.38 (±27.3)	0.2190
Grafts/patient	3.7 (±0.9)	3.7 (±0.8)	0.3510
Priority			
Elective	23.2%	24.6%	0.6636
Urgent	72.8%	69.5%	0.1670
Emergent	3.8%	5.6%	0.2230
Permanent stroke	1.2%	1.2%	1.0000
Perioperative MI	0.50%	0.99%	0.4930
Sternal wound infection	1.5%	3.2%	0.0785
Septicemia	1.5%	1.7%	0.8200
Reoperation for bleeding	1.5%	2.2%	0.4570
Respiratory failure	3.0%	3.2%	0.8690
Renal failure	1.3%	1.5%	0.8080
Hemoglobin A1C	7.62%	7.99%	0.6080

**Figure 1:**
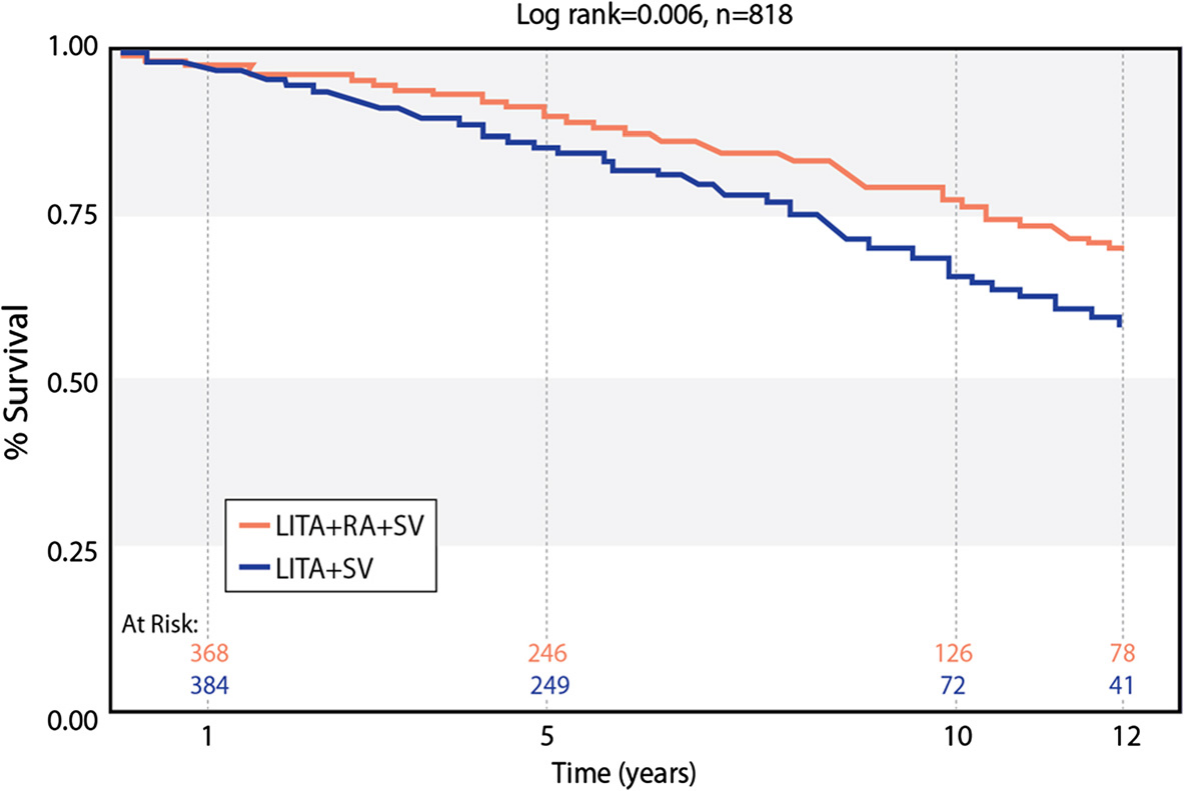
Kaplan-Meier survival curve of propensity matched 818 diabetic patients after CABG.

After propensity matching, postoperative major complications occurred with equal frequency in each of the two groups as summarized in Table 4. (Although sternal wound infection was higher in SV this was not statistically significant). There were a total of 77 late deaths (18%) in the RA group and 104 deaths (25.4%) in the SV group; p=0.023. The average time to death was 6.2 ±3.5 years (median 5.8 years) for the RA patients and 5.8± 3.5 years (median 6.0 years) for the SV patients. We were not able to differentiate cardiac from other causes of death. Kaplan Meier actuarial survival curves derived for RA and SV groups are depicted in Figure 1. Survival at 1, 5, 10 and 12 years was 98%, 89%, 77 and 70% for RA vs. 96%, 87%, 64% and 59% for SV and this was highly statistically significant by Log Rank Test (p = 0.006).

Cox multivariable analysis determined independent risk factors predictive of mortality, and these are listed in Table 5. As expected, age exerts a significant difference per year and ejection fraction is inversely linked to mortality. Stroke, peripheral vascular disease and renal dysfunction exert a strong influence, almost doubling mortality. There is only a single factor statistically protective against mortality for diabetic patients: use of the RA with hazard ratio 0.683 (confidence intervals 0.507 - 0.920, p < 0.0122), suggesting a greater than 30% risk reduction in mortality attributed to use of the RA in diabetic patients undergoing CABG.

**Table 5:** Independent predictors of mortality (Cox Method) 818 matched diabetic patients

	**Hazard ratio**	**95% C I**	**P value**
Stroke	1.920	(1.257-2.935)	0.0026
Femoral PVD	2.363	(1.688-3.307)	<0.0001
Creatinine > 2.5	2.196	(1.114-4.328)	0.0231
Age (per year)	1.056	(1.036-1.075)	<0.0001
EF (per % point)	0.980	(0.969-0.992)	0.0007
Radial Artery Use	0.683	(0.507-0.920)	0.0122

## Discussion

While diabetes is increasing at an alarming rate, coronary artery disease (CAD) is more prevalent among diabetics and more severe, generally more diffuse, extensive and often more distally distributed [8]. Since the BARI trial and to the present, as reflected in current ACC/AHA guidelines [9], compelling evidence supports referral for surgical revascularization for diabetic patients with multi vessel CAD. Importantly, however, even as survival has improved for all groups, survival of diabetic patients remained worse than non diabetic patients immediately after surgical revascularization and at every stage thereafter [10]. Several specific factors affect diabetics: increased platelet aggregation, increased platelet adhesion, and increased thrombogenesis likely contribute to premature graft failure and the increase in postoperative myocardial ischemic events [11]. Outcomes are linked to pre-operative control of blood sugar reflected by Hemoglobin A1C [12]. Naturally, the increased incidence of peripheral vascular disease in diabetes might impact complications associated with harvesting of the conduit [13].

RA is a versatile arterial bypass conduit: easily harvested, of appropriate size and easy to handle. In our experience, RA harvest complications were vanishingly rare: forearm infections were extremely rare and major nerve injury did not occur; no patient has any reported or measurable functional deficit. Choudhary [14] identified augmented radial vasoreactivity in diabetics compared with non-diabetics, but the clinical significance is unclear. Distensibility and compliance of RA are not reduced in diabetics but radial intima-medial thickness and wall cross sectional area are significantly higher [15]. Visibly calcified or atherosclerotic RA conduit was discarded but these circumstances were very rare.

Long term SV patency is rather disappointing in most reported series. Superior patency of the LITA and the impact on long term survival is universally acknowledged. Right ITA patency has also been shown to be superior to SV [3], but the survival advantage of bilateral ITA grafting has not been confirmed in all studies. In studies focusing specifically on diabetic patients, while Lev-Ran [16] reported survival and MACE benefits for bilateral ITA grafting in patients on oral diabetic medicines, Hirotani found no advantage for bilateral ITA in diabetics at 10 years. Excellent graft patency was documented early after CABG. In many of their patients the RITA was used to bypass the LAD and the LITA to a lateral wall vessel, a strategy which makes subsequent reoperative sternotomy hazardous [17]. Toumpoulis [18] also reported no survival advantage for diabetic patients at 5 years for bilateral versus single ITA grafting: it may be that their follow up period was too short to detect the long term patency advantages of multiple arterial grafts. By contrast, however, RAPS [19] reported RA benefit for diabetics based on a patency rate greater than SV on angiography at one year. Major concerns persist, especially in diabetics, about the impact of bilateral ITA harvest on sternal healing and infection [20]. So it is perhaps not surprising that, despite the strong evidence in favor of bilateral ITA use (only some of which is cited above) the STS database records a disappointingly low (4%) use of bilateral ITA for CABG [21].

We, and others in the USA, Canada, Europe and Australia [2,3,5,18] have documented excellent mid-term patency of RA grafts. Guidelines [9] list radial use for CABG as a Class IIb recommendation: *“ Use of a radial artery graft may be reasonable when grafting left-sided coronary arteries with severe stenoses (>70%) and right-sided arteries with critical stenoses (90%) that perfuse LV myocardium. (Level of Evidence: B )”* Despite encouraging outcomes, RA is used in only 9% of CABG patients in the STS database [21]. Acar [22] recently published a remarkable appraisal of a personal 20 year experience in 819 RA patients of whom a third (32%) were diabetic. The right coronary artery was the target for the RA graft in 30% of cases, and would likely not have met the current guidelines! Conventional angiography or CT was used to assess almost half the patients (351) a mean of 7 years after CABG in symptomatic and asymptomatic patients with no difference in patency between SV and RA (82% vs 81.9%). Beyond the first year after CABG the RA patency rate was steady out past 13 years. Notably, patients in his series received on average just 2.45 grafts each (his series was not limited to patients operated on for first time coronary bypass surgery alone), and only veins of “faultless” quality were used, which likely explains the extraordinarily high SV patency in his series.

The Toledo group [23] identified a survival benefit for RA use in young diabetics with triple vessel disease from their patient cohort of whom 34% were diabetics. However, in a subsequent report [24], they were unable to demonstrate a survival benefit for RA grafting in 950 propensity matched diabetic patients, separated into insulin and no-insulin categories. They did not have data on graft patency rates, or cause of death, and included operative deaths in their analysis. Late mortality for their RA patients was higher than in our experience, and their reported mortality was similar to that in our SV patients. With a shorter follow up interval than ours, they had only 28 RA patients at risk at 10 years. In many ways our experience parallels theirs, and it is quite striking that the relative risk reduction afforded by RA use in their unmatched patients (n=566, 151 of 626 RA compared with 415 of 890 SV) analyzed after risk adjustment is remarkably similar (30%) to the 32% we found for our 818 propensity matched patients. However while their comparison of unmatched but risk adjusted groups failed to reach statistical significance (95% CI 0.36 -1.37; p= 0.29) our propensity matched comparison of larger RA and SV groups showed the 32% relative risk reduction achieved in our RA group to be highly statistically significant and therefore RA use in our series statistically predicts better survival (HR 0.683, CI 0.507- 0.920, p=0.0122).

Addressing concerns about patency of endoscopically harvested saphenous veins [25], our experience provides comforting evidence that endoscopic harvest does not compromise graft patency or patient survival. Despite improved systems for endoscopic harvest, it must be emphasized that the level of technical proficiency required to harvest the RA arterial conduit is substantial and remains highly operator dependant.

### Limitations

Although our analysis is retrospective, preoperative, operative and in-hospital postoperative data are derived from a mandatory, audited state database and were collected and recorded prospectively. Only isolated primary CABG patients were analyzed, and this series represents the largest single site consecutive experience with standardized techniques. Survival data are derived from the Social Security Death Index with its known imperfections. Although a propensity match is considered the appropriate statistical tool to compare outcomes of different treatments, this may not adjust fully for selection bias (surgeon choice) and patient heterogeneity.

## Conclusion

In diabetic patients who have coronary revascularization with LITA to the LAD, the addition of a radial artery graft produces a significant and sustained improvement in long-term survival, when compared with LITA and SV grafting. This reinforces our hypothesis that use of the radial conduit can overcome the previously universally acknowledged worse long-term outlook for diabetic patients after coronary bypass surgery. With these data, optimal revascularization for diabetic patients is better defined.

### Implications

Our findings have important implications for planning the initial revascularization strategy for every diabetic patient with multivessel coronary disease.

## Abbreviations

PCI: Percutaneous coronary intervention; CABG: Coronary artery bypass grafting; RA: Radial artery; SV: Saphenous vein; LITA: Left internal thoracic artery; RITA: Right internal thoracic artery; COPD: Chronic obstructive pulmonary disease; LAD: Left anterior descending; MI: Myocardial infarction; PVD: Peripheral vascular disease; CHF: Congestive heart failure; RAPS: Radial artery patency study.

## Competing interests

The authors declare that they have no competing interests.

## Authors’ contributions

DMH AND RFT conceived of the study, and lead study design and coordination. DMH drafted and RFT helped to draft the manuscript. WK and CMG participated in study design and manuscript revisions. HDeC collected the data. KRD participated in the design of the study, supervised data collection and with PF performed the statistical analysis. All authors read and approved the final manuscript.
